# A rational use of glucocorticoids in patients with early arthritis has a minimal impact on bone mass

**DOI:** 10.1186/ar2961

**Published:** 2010-03-23

**Authors:** Monica Ibañez, Ana M Ortiz, Isabel Castrejón, J Alberto García-Vadillo, Inmaculada Carvajal, Santos Castañeda, Isidoro González-Álvaro

**Affiliations:** 1 Rheumatology Department, Hospital Son Llàtzer, Carretera Manacor km. 4, Palma de Mallorca, 07198, Spain; 2 Rheumatology Department, Hospital Universitario de La Princesa, Diego de León 62, Madrid, 28006, Spain; 3 Rheumatology Unit, Hospital Nuestra Señora del Rosario, Príncipe de Vergara 53, Madrid, 28006, Spain

## Abstract

**Introduction:**

Glucocorticoid (GC)-induced osteoporosis is a frequent complication in patients with rheumatoid arthritis. However, little information exists about the consequences of GC use in patients with early arthritis. Here we describe the variables underlying the use of GC in early arthritis, as well as its effect on bone-mineral density.

**Methods:**

Data from 116 patients in our early arthritis register were analyzed (90 women; median age, 52.5 years, interquartile range (IQR, 38.5-66); 6-month median disease duration at entry (IQR, 4-9)). In this register, the clinical and treatment information was recorded systematically, including the cumulative GC dose. Lumbar spine, hip, and forearm bone-mineral density (BMD) measurements were performed at entry and after a 2-year follow-up. A multivariate analysis was performed to establish the variables associated with the use of GCs, as well as those associated with variations in BMD.

**Results:**

Of the patients with early arthritis studied, 67% received GCs during the 2-year follow-up. GCs were more frequently prescribed to elderly patients, those with higher basal disease activity and disability, and patients with positive rheumatoid factor. When adjusted for these variables, GCs were less frequently prescribed to female patients. The use of GCs was associated with an increase of BMD in the ultradistal region of the forearm, although it induced a significant loss of BMD in the medial region of the forearm. No relevant effect of GC was noted on the BMD measured at other locations.

**Conclusions:**

The frequent use of GCs as a "bridge therapy" in patients with early arthritis does not seem to be associated with relevant loss of bone mass. Moreover, cumulative GC administration might be associated with an increase of juxtaarticular BMD.

## Introduction

Rheumatoid arthritis (RA) is a systemic and chronic inflammatory disease that has been associated with disability, the existence of comorbidities, and decreased life expectancy [1,2]. The use of glucocorticoids (GCs) to treat RA offers rapid antiinflammatory effects and the capacity to arrest radiologic progression [3-6]. However, long-term GC use may cause multiple adverse events, even at low doses [7,8]. Therefore, an individual evaluation is required to establish the best risk/benefit ratio for their prescription [9,10].

One of the most striking side effects of this drug is GC-induced osteoporosis (GIOP), a complication in patients with RA that can be prevented [11-13]. The prevalence of OP in RA patients is higher than that in the normal population, ranging from 20% to 37% [14,15], although this figure increases to almost 50% among postmenopausal women after long-term steroid use [16]. However, establishing the real contribution of GCs to OP in RA is challenging because bone mineral loss is of multifactorial origin in these patients, and it may be influenced by inflammatory cytokines, inactivity, GCs, disease-modifying antirheumatic drugs (DMARDs), as well as the classic risk factors for OP.

Despite the different studies focusing on OP, little information is available regarding the use of GCs in patients with early arthritis. Nevertheless, it has been suggested that using GC in these patients does not affect bone mass, as has been observed in the long-term disease, probably because controlling inflammatory activity at early stages may prevent bone loss [17-19].

The aim of this study was to analyze the patterns of GC use and the reasons for its use in a population of early arthritis patients. In addition, we studied the impact of different factors on the evolution of mineral bone content in these patients, including GC use.

## Materials and methods

### Patients and study design

We studied 116 patients who attended our Early Arthritis Clinic from July 2002 to April 2008. Patients were referred to the clinic if they had two or more swollen joints for ≥ 4 weeks and symptoms for <1 year. Patients were excluded if diagnosed with gouty arthritis, septic arthritis, spondyloarthropathies, or connective tissue diseases during the follow-up period. In addition, we excluded patients with primary hyperparathyroidism and other metabolic bone disorders other than OP. At the end of the follow-up period, 78 patients fulfilled the ACR 1987 criteria for RA classification [20], and 38 patients were classified as having undifferentiated arthritis (UA). The characteristics of the patients are shown in Table 1. The study protocol was reviewed and approved by the Local Research Ethics Committee, and all the patients who entered the study signed a written consent form after being informed about the details of the protocol.

**Table 1:** Baseline population characteristics

	Total (n = 116)	RA (n = 78)	UA (n = 38)	*P*
Age	52.5 [38.5-65.6]	51.6 [43.0-66.7]	53.5 [34.0-64.5]	NS
Female gender, *n *(%)	90 (77.6)	63 (80.7)	27 (71.1)	NS
Disease duration (mo)	6.1 [4.2-9.1]	5.6 [4.0-8.4]	6.75 [4.5-9.8]	NS
HAQ	1.0 [0.5-1.5]	1.1 [0.5-1.6]	0.8 [0.5-1.3]	NS
DAS28	3.9 [3.2-5.2]	4.5 [3.6-5.8]	3.3 [2.8-4.6]	0.001
Rheumatoid factor, *n *(%)	45 (38.8)	38 (48.7)	7 (18.4)	0.002
Anti-CCP, *n *(%)	39 (33.6)	36 (46.1)	3 (7.9)	<0.001

Comorbidities: *n *(%)				
Hypertension	35 (30.2)	22 (28.2)	13 (34.2)	NS
Diabetes mellitus	13 (11.2)	8 (10.2)	5 (13.2)	NS
Thyroid dysfunction	17 (14.6)	15 (19.2)	2 (5.2)	0.1

Menopause (% of women)	58	59	56	NS
Age at menopause (years)	50 [44-52]	48 [44-50]	52 [50-53]	0.017
Calcium intake (rare/moderate/high), (%)	15/62/23	14/65/21	16/58/26	NS
Smoking *n *(%)	28 (24.1)	19 (24.4)	9 (23.7)	NS
Exercise (rare/moderate/intense) (%)	38/47/15	42/42/16	29/58/13	NS
Body mass index (kg/m^2^)	26.5 [24.5-30.5]	26.0 [24.0-30.0]	28.0 [26.0-30.9]	NS
Prior personal fractures (%)	17	18	16	NS
Prior family fractures (%)	19	18	24	NS

Data are shown as the percentage of patients or as the median and interquartile range.

Abbreviations: anti-CCP, antibodies directed to cyclic citrullinated peptide; DAS28, disease activity score in 28 joints; HAQ, health assessment questionnaire; NS, not statistically significant; RA, rheumatoid arthritis; UA, undifferentiated arthritis.

The study includes data from patients followed up over a period of 2 years and who were evaluated at four visits during this period. The following data were collected and entered into an electronic database: clinical and demographic information, including the 28 tender and swollen joint counts (TJCs and SJCs, respectively); global disease assessment by patient (GDAP) and physician (GDAPh) on a 100-mm visual analogue scale; and basic laboratory tests including erythrocyte sedimentation rate (ESR), C-reactive protein (CRP), rheumatoid factor (RF: by nephelometry; positive if >20 IU/ml), and serum antibodies directed against cyclic citrullinated peptide (anti-CCP: ELISA, Euro-Diagnostica Immunoscan RA; positive at >50 IU/ml). The four-component disease-activity score based on 28-joint counts and ESR (DAS28) was calculated as described previously [21]. The patients also completed the validated Spanish version of the Health Assessment Questionnaire (HAQ) to assess functional ability [22].

Comorbidity of other medical conditions was evaluated during the follow-up period, including that of hypertension, diabetes mellitus, and thyroid dysfunction. Other known factors that affect bone mass were also assessed, such as body mass index (BMI) expressed in kilograms per square meter, age at menopause, daily calcium intake (0-500 mg, 500-1,000 mg, or >1,000 mg), exercise (sedentary, moderate, or intense aerobic exercise), smoking, and clinical fractures (vertebral, peripheral, or both) before inclusion in our register and during the follow-up period.

Information about disease-modifying antirheumatic drugs (DMARDs) treatment during the follow-up period, the dose of prednisone at each visit, and the cumulative GC dose (as a prednisone equivalent) also were obtained. Regarding the latter, we separately collected the cumulative dose of GCs prescribed orally and that administered as joint and soft-tissue injections (see Additional file 1 for further information). Most injections were performed in the knee or shoulder; no wrist injections were performed, although a few injections were administered into the small joints of the hand, mainly proximal interphalangeal.

### Bone-mineral density measurement

Dual-energy x-ray absorptiometry (DXA) scans were performed on a Hologic QDR-4500/W Elite densitometer (Hologic Inc., Bedford, MA, USA), and the bone mineral density (BMD) was expressed in grams per square centimeter. Lumbar spine, hip, and nondominant forearm DXA scans were carried out at the patient's first (median disease duration, 7 months (IQR, 4-9)) and last visit in the study (median disease duration, 32.5 months (IQR, 29-35)). The densitometer was calibrated daily by using a quality assurance spine phantom of known bone mineral content (BMC) supplied by the manufacturer. The *in vivo *short-term coefficient of variation for our DXA machine was 0.9% for measurements at the lumbar spine level and 1.4% for the duplicate total hip measurements in 10 healthy subjects (data not shown).

We calculated the yearly variation in BMD at each location as follows: ΔBMD = (BMD_final _- BMD_baseline_) × 365/number of days between both measurements. The results of these variables are presented as milligrams per square centimeter per year.

### Statistical analysis

The descriptive analysis was performed by calculating the means and standard deviations (SDs) of quantitative variables with a gaussian distribution. The median and the interquartile range (IQR) were calculated if the variables did not display a normal distribution. An estimate of the proportions was calculated for qualitative variables. Unless otherwise stated, Student's *t *test was applied to compare the means of variables with a normal distribution, and the Mann-Whitney or Kruskall-Wallis tests were used for variables that did not have a normal distribution. Fisher's test was used to compare the categoric variables.

Because one third of the patients did not take GCs during the follow-up period, we used the *zip *command of Stata 9.2 for Windows (StataCorp LP, College Station, TX, USA) to analyze the cumulative dose of GCs. This command defines a zero-inflated Poisson regression that enables us to analyze both the reasons underlying the zero counts (no GCs prescribed) and those associated with the cumulative GC dose. All variables associated with a *P *≤ 0.15 in the bivariate analysis were included as independent variables, both to estimate the Poisson regression of the dependent variable (cumulative GC dose in milligrams per month) and in the *inflate *option of the *zip *command. This option specifies the equation that determines whether the observed count is zero. The final model was then reached by using stepwise backward estimates, removing all variables with *P *> 0.15.

A generalized linear model was applied to assess the independent effect of different variables on the ΔBMD at the lumbar spine, hip, and forearm. We used the *glm *command of Stata 9.2 to define the linear regression of all the variables with a *P *value ≤ 0.15. The final model was then reached by using stepwise backward estimations, removing all variables with *P *> 0.15. Subsequently, the cumulative GC dose was forced into the model to determine whether it affected the variation in BMD once the model was adjusted for the variables considered relevant. To assess whether oral and intraarticular/soft tissue injection had equivalent effects on BMD, we also developed a model with two independent variables, one for the cumulative GC dose prescribed orally and another for the cumulative GC dose administered as soft-tissue and joint injections. However, this model did not provide more information than the model that included the effect of the global cumulative GC dose.

## Results

### Description of the use of glucocorticoids

**Figure 1:**
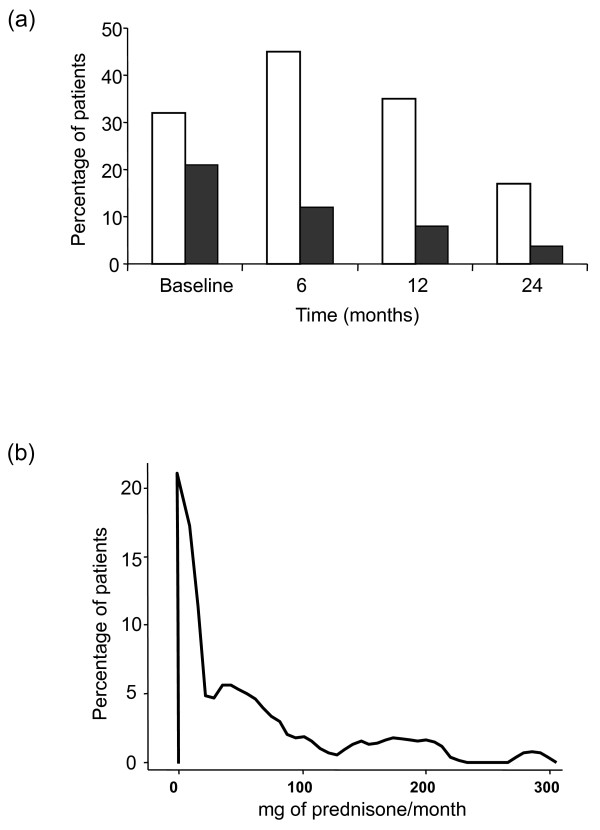
**Use of glucocorticoids (GCs) in patients with early arthritis**. **(a) **Percentage of patients that received GCs at each follow-up visit. White columns represent the percentage of patients receiving GCs; black columns represent the patients who received >7.5 mg of GC per day. **(b) **Distribution of cumulative GC dose adjusted to the duration of the follow-up in the whole population. The graph was obtained by using the *kdensity *command of Stata 9.2, which provides kernel density estimates of continuous variables in a population.

Among our 116 patients, 38 (32.7%) received GCs orally, 11 (9.4%) received only soft-tissue injections, and 28 (24.3%) were administered GCs by both routes during the follow-up period. Oral GC therapy was prescribed mostly as a bridging therapy, and it commenced at the first visit (Figure 1a), although about 11% of patients received the drug later in the follow-up. Thus, the prescription of GCs increased by 45.4% during the first 6 months, and then it gradually decreased to only 17.3% by the end of the follow-up period (Figure 1a). Doses of prednisone >7.5 mg/day were prescribed to 21.5% of patients at the baseline, although by the end of the study, only 3.7% of patients still received such high doses (Figure 1a and Additional file 2). The median cumulative GC dose in the whole population during the study was 865 mg (IQR, 0-2,263), and when adjusted for the duration of the follow-up, it was 22 mg/month (IQR, 0-70) (Figure 1b). When only the patients that received GCs were considered, the median cumulative dose of GCs was 1,656 mg (855-3,751) and 45 mg/month (IQR, 21-106) when adjusted to the duration of the follow-up. More-extensive information on the use of GCs in our population is provided in Additional file 2.

The prescription of GCs was associated with male gender and the more-advanced age of the patients. In addition, a tendency was noted to prescribe GCs more frequently to those patients with a higher baseline DAS28 and HAQ, although this was not statistically significant in our cohort. In those patients who received GCs, the cumulative dose was significantly higher in men, in older patients, in those patients with seropositive arthritis or a worse functional status, in those with a higher disease activity at baseline, as well as in those treated with combined DMARD therapy. An inverse relation between cumulative GC dose and disease duration at baseline was also observed. Once adjusted for all these variables, patients with UA received higher doses of prednisone than did those with RA. More-extensive information on the motives underlying GC prescription and the cumulative GC dose is provided in Additional file 3.

### Effect of glucocorticoids on bone mass

**Figure 2:**
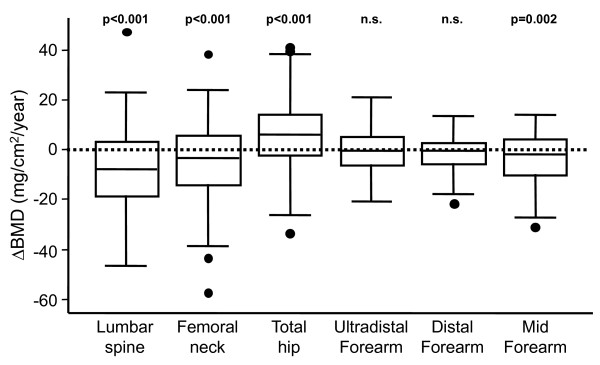
**Evolution of bone-mineral density (BMD) in patients with early arthritis**. Variation of BMD (ΔBMD) at each site was calculated as described in the Patients and Methods sections. Dotted horizontal line represents the absence of variation in BMD. Data are presented as the interquartile range (p75 upper edge of the box, p25 lower edge, p50 midline in the box), as well as the p95 (upper line from the box) and p5. Dots represent the outliers. Statistical significance was established through the Wilcoxon test for paired samples.

During the follow-up period, generally a significant decrease in BMD was noted at all sites, except for a significant increase in BMD detected in the total hip measurement. No significant variations were detected at the ultradistal and distal forearm (Figure 2).

Although we observed slight differences in the factors associated with the variation in bone mass at different locations, the variables that were globally associated with a significant decrease in BMD were menopause, diabetes, and thyroid disorders (Table 2). In addition, patients with higher disease activity displayed a trend toward a greater decrease in BMD at the lumbar spine, total hip, and ultradistal forearm, and the association of the mean DAS28 with BMD loss during follow-up was significant at the lumbar spine (Table 2). By contrast, BMD at the lumbar spine and the total hip BMD increased significantly in older patients (Table 2), which was probably related to osteoarthritis, as described previously [23,24].

**Table 2:** Variables associated with the variation in bone-mineral density (mg/cm^2^/year) at different sites in patients with early arthritis

	Multivariate analysis									
	
	Lumbar spine		Total hip		UD forearm		D forearm		M forearm	
					
	Coeff ± SD	*P*	Coeff ± SD	*P*	Coeff ± SD	*P*	Coeff ± SD	*P*	Coeff ± SD	*P*
Age (by year)	0.3 ± 0.1	0.006	0.2 ± 0.1	0.026	-	NI	-	NS	-	NI
Female gender	-	NS	6.1 ± 3.6	0.094	8.7 ± 4.1	0.032	-	NI	-	NS
Menopause	-	NI	-	NI	-6.4 ± 3.3	0.057	-3.5 ± 1.4	0.01	-	NI
Hypertension	6.9 ± 3.9	0.081	-	NS	-	NI	-	NS	6.7 ± 3.6	0.06
Diabetes	-19.5 ± 4.8	<0.001	-	NI	-	NI	-	NI	-10.3 ± 5.3	0.05
Thyroid dis.	-13.7 ± 3.9	<0.001	-	NI	-10.3 ± 4	0.01	-	NI	-	NI
Mean DAS28	-0.3 ± 0.1	0.042	-0.2 ± 0.1	0.141	-2.3 ± 1.6	0.145	-	NS	-	NI
GC use (mg/mo)	0.01 ± 0.02	NS	-0.01 ± 0.02	NS	0.05 ± 0.02	0.024	-0.001 ± 0.009	NS	-0.05 ± 0.02	0.025

Abbreviations: D, distal; DAS28, disease activity score in 28 joints; M, medium; NS, not statistically significant; NI, not included in the analysis; Thyroid dis., thyroid disorders; UD, ultradistal.

**Figure 3:**
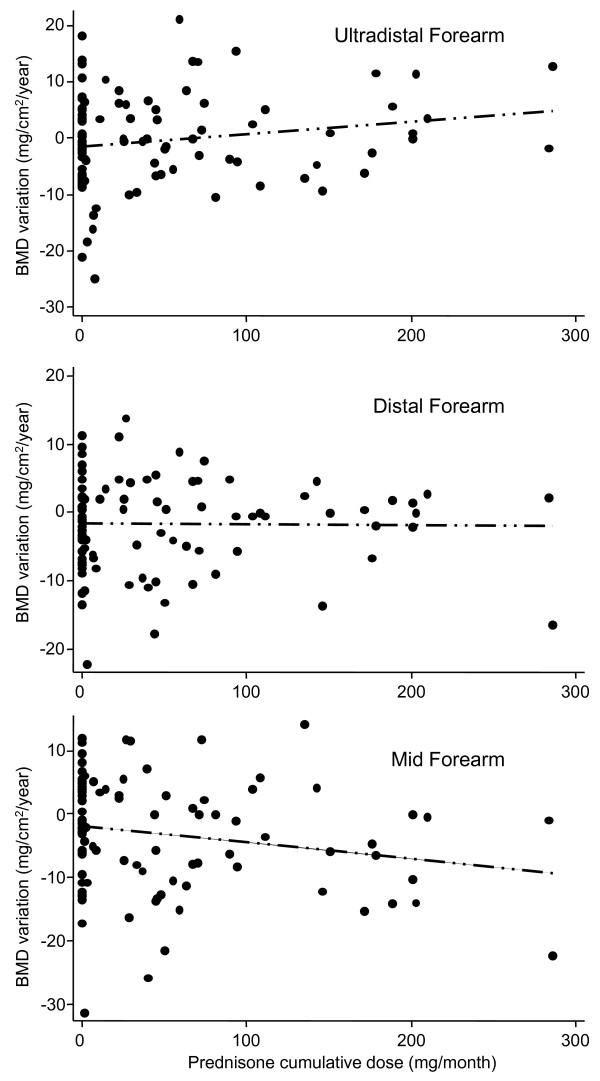
**Correlation between cumulative doses of GC (mg/month) and the annual variation in BMD (mg/cm^2^/year)**. **(a) **ultradistal, **(b) **distal, and **(c) **mid-forearm. Data are shown as dot plots and the estimated linear regression (dotted line).

Interestingly, we observed a positive correlation between the cumulative dose of GCs and the variation in BMD at the ultradistal forearm (Figure 3, upper panel; *r *= 0.22, *P *= 0.08), although these parameters were negatively correlated at the mid-forearm (Figure 3, lower panel; *r *= -0.2; *P *= 0.11). These findings were statistically significant in the multivariate analysis after adjustment for the independent variables described earlier (Table 2). Conversely, the cumulative dose of GCs did not seem to influence BMD significantly at the other sites analyzed (Table 2).

## Discussion

The most intriguing finding in our study was that the use of GCs has no relevant impact on bone mass in patients with early arthritis. Earlier studies that focused on the effect of GCs on bone mass in patients with early arthritis maintained fixed low doses of GC for long periods, or alternatively, they involved a schedule that tapered the doses of these drugs from high to low doses and then withdrew the GC, or maintained it at low doses for a long period [18,25]. However, in our early-arthritis clinic, no preestablished therapeutic protocol existed, and GCs were prescribed mainly as a bridging therapy in patients with more-severe forms of the disease, on average starting at 15 mg/day of prednisone and then gradually tapering to its withdrawal ~14 months later. No more than 50% of patients received GCs orally, and only 17.3% of patients were under treatment with GCs at the end of the study. In addition, the prescription of the drug was adjusted to the profile of comorbidity, the disease severity, and the patients' preferences. Thus, the prescription of GCs was more frequent in older patients (possibly related to the tendency to be less aggressive with DMARDs because of their higher comorbidity) and male patients (independent of age and disease activity), probably because women are more worried about the cosmetic side effects of GCs, and they reject high doses or long steroid treatments.

Unexpectedly, after adjustment for confounding factors in the multivariate analysis, patients with UA received more cumulative doses of GCs than did RA patients. This may reflect the preference in our center to use GCs instead of DMARDs in patients who do not meet ACR criteria for RA, at least during the first months of the follow-up.

The main variables associated with bone loss in patients with early arthritis are similar to those affecting the general population: the menopause and comorbidities such as diabetes or thyroid disorders. Interestingly, despite the limited number of patients in our study, we could detect an association between disease activity and bone loss in the lumbar spine, ultradistal forearm, and the total hip measurements, as suggested previously [26]. However, we did not find any relation between the annual variation of BMD and other factors such as calcium intake, exercise, smoking, personal or family history of fractures, or BMI (data not shown). The failure to demonstrate such a relation may reflect the limited number of patients, or perhaps, these factors may have less weight in the variation in bone mineral content in patients with arthritis than among the general population.

Intriguingly, a trend was noted toward higher bone mineral content in patients with hypertension at two of the sites where bone mass was measured. This increase might be related to the relatively high use of thiazides in association with renin-angiotensin antagonists in patients with hypertension in our country [27], particularly given that both kinds of drugs have been associated with improvements in BMD [28-31].

In accordance with previous studies in early arthritis, we did not find a significant correlation between cumulative doses of GCs and BMD variation at the lumbar spine or hip [18,19,26]. Nevertheless, our most relevant finding was at the forearm, where BMD has not previously been evaluated. We observed a clear association of the cumulative GC dose with an increase in BMD at the ultradistal forearm. This finding is probably related to the rapid and strong control of inflammation by GCs and most likely to its ability to arrest osteoclast function [32]. In this regard, GIOP was recently proposed to be mediated through the upregulation of a receptor activator for nuclear factor κB ligand (RANKL) expression and the inhibition of osteoprotegerin expression [33-35]. However, with regard to RA synovitis, the levels of multiple cytokines with osteoclast-inducing activity, including RANKL, are elevated [36], and intraarticular GC decreases synovial RANKL expression [37]. This latter finding may be related to the increase in juxtaarticular BMD described here, which might also be associated with the ability of GC to impair joint destruction in early RA [25].

By contrast, moderate bone loss was found at the mid-forearm, where 95% is cortical bone. It is tempting to hypothesize that these effects might be explained by the development of secondary hyperparathyroidism due to the influence of GC on calcium metabolism [38,39]. Indeed, three patients had symptomatic fractures during follow-up (Additional file 4), two of which were peripheral fractures associated with cortical weakness.

One possible limitation of our study is the limited number of patients studied. However, we think that this deficit can be balanced by the exhaustive data collected in a highly controlled population. In addition, BMD was evaluated in six different anatomic locations. Thus, if we found a weak association at several locations, we could assume that it would have been less likely to have occurred by chance. It might also be argued that 2 years is a short period in which to study variations in BMD. However, it is well known that the effect of GC on bone mass occurs soon after exposure. It would have been interesting to measure BMD every 6 months during the follow-up period, although our schedule of BMD assessment was based on the follow-up recommendations for osteoporotic patients [40].

## Conclusions

Among our patients with early arthritis, GCs were prescribed mainly as a bridge therapy to elderly patients, men, and patients with severe forms of the disease. This pattern of use, starting on average with 15 mg/d of prednisone and tapering toward withdrawal about 1 year later, does not seem to represent a relevant risk factor for bone loss. Furthermore, the cumulative GC dose correlated with an increase of juxtaarticular BMD, once adjusted for the classic variables associated with primary OP.

## Abbreviations

anti-CCP: serum antibodies directed to cyclic citrullinated peptide; BMD: bone-mineral density; DMARDs: disease-modifying antirheumatic drugs; GCs: glucocorticoids; GIOP: glucocorticoid-induced osteoporosis; OP: osteoporosis; RA: rheumatoid arthritis; RANKL: receptor activator for nuclear factor κB ligand; RF: rheumatoid factor.

## Competing interests

Over the past 5-year period, Dr. I. González-Álvaro has received unrestricted research funds from Abbott Laboratories, Sanofi-Aventis, and Bristol-Myers Squibb. All these research projects bear no relation to this work.

## Authors' contributions

MI participated in the acquisition and interpretation of the data and drafted the manuscript. AMO and I Castrejon participated in the data acquisition and helped to draft the manuscript. AG-V and SC participated in the design of the study and helped to draft the manuscript. I Carvajal participated in the data acquisition. IG-A participated in the design of the study and in the data acquisition, in the statistical analysis, in the interpretation of the data, and helped to draft the manuscript. All authors read and approved the final version of the manuscript submitted.

## Supplementary Material

Additional file 1 **Intraarticular or soft-tissue glucocorticoid injections: equivalencies in milligrams of prednisone**. To calculate cumulative dose of glucocorticoids, doses corresponding to intraarticular and soft-tissue injections were estimated in milligrams of prednisone according to this table. Click here for file

Additional file 2 **Prescription of glucocorticoids in the population of early arthritis patients**. These two tables provide detailed information regarding the characteristics of the population depending on the prescription of glucocorticoids and how this drug was used. Click here for file

Additional file 3 **Variables associated with glucocorticoid prescription and the cumulative dose of this drug by month of follow-up**. Multivariate analysis that provides information about the variables that explain GC prescription and those associated with the cumulative dose of GC. Click here for file

Additional file 4 **Prescription of drugs for osteoporosis and the incidence of fractures during the follow-up**. This file provides information about prevalence and variables associated with the prescription of drugs for osteoporosis. In addition, it describes the incidence of clinical fractures in the population described in the article. Click here for file
