# Supplementary material for: A rational use of glucocorticoids in patients with early arthritis has a minimal impact on bone mass
Source: Arthritis Res Ther. 2010 Mar 23;12(2):R50. doi: 10.1186/ar2961 (PMC2888199; doi:10.1186/ar2961)
Supplement: Additional file 1 — Intraarticular or soft-tissue glucocorticoid injections: equivalencies in milligrams of prednisone. To calculate cumulative dose of glucocorticoids, doses corresponding to intraarticular and soft-tissue injections were estimated in milligrams of prednisone according to this table. [file ar2961-S1.DOC]

# Additional Table 1. Intra-articular or soft tissue glucocorticoid injections. Equivalencies in mg of prednisone

| Depot glucocorticoid | |
| --- | --- |
| Celestone Chronodose® (bethametasone 12 mg) | 50 mg of prednisone |
| Trigon depot ® (triamcinolone acetate 40 mg) | 80 mg of prednisone |
| According to site | |
| Large joints and tendons | 65 mg of prednisone |
| Small joints and tendons | 25 mg of prednisone |

When the type of glucocorticoid used was not specified in the clinical records, we considered the mean of one vial of Celestone Chronodose and one injection of trigon depot (65 mg of prednisone) when injected into the shoulders, knees or the trochantereal bursa. When injected into small joints, carpal tunnel or small tendons, we considered 25 mg of prednisone.
