# Supplementary material for: A rational use of glucocorticoids in patients with early arthritis has a minimal impact on bone mass
Source: Arthritis Res Ther. 2010 Mar 23;12(2):R50. doi: 10.1186/ar2961 (PMC2888199; doi:10.1186/ar2961)
Supplement: Additional file 2 — Prescription of glucocorticoids in the population of early arthritis patients. These two tables provide detailed information regarding the characteristics of the population depending on the prescription of glucocorticoids and how this drug was used. [file ar2961-S2.DOC]

Additional Data 2. Prescription of glucocorticoids in the population of Early Arthritis Patients Additional Table 2A

| Glucocorticoids | Oral (n=38) | Oral + IAI (n=28) | IAI (n=11) | None (n=36) |
| --- | --- | --- | --- | --- |
| Female gender (%) | 65.7 | 73.1 | 90 | 83.8 |
| Age (y-o) | 61 [45 – 70] | 57 [43 – 69] | 51 [45 – 55] | 42 [31 – 54] |
| GC cumulative global dose (mg) | 2169 [953 – 3970] | 1614 [1041 – 3751] | 65 [25 – 180] | - |
| GC cumulative oral dose (mg) | 1774 [865 – 3623] | 1048 [544 – 1950] | - | - |
| GC cumulative IA dose (mg) | - | 140 [65 – 200] | 65 [25 – 180] | - |
| Max. oral GC dose | 15 [20 – 10] | 15 [20 – 10] | - | - |
| Min. oral GC dose | 0 [0 – 1.3] | 0 [0 – 1.3] | - | - |
| Time with oral GC (months) | 17 [10 – 25] | 11 [6 – 23] | - | - |
| Number of IAI | - | 2 [1 – 4] | 1 [1 – 3] | - |

Abbreviations: n: number; IAI: intra-articular injections; y-o: years old; GC: glucocorticoid.

Additional Table 2B

|  | | Visit 1  n=116 | Visit 2  n=102 | Visit 3  n=106 | Visit 4  n=116 |
| --- | --- | --- | --- | --- | --- |
| Patients with GC | RA (%) | 42 | 55 | 33 | 20 |
|  | UA (%) | 11 | 21 | 16 | 11 |
|  | Total n (%) | 37 (32) | 46 (45) | 29 (27) | 20 (17) |
| GC ≥7.5 mg/d | RA (%) | 28 | 16 | 10 | 3 |
|  | UA (%) | 8 | 4 | 3 | 5 |
|  | Total n (%) | 25; 21.5 | 13; 13 | 8; 7.5 | 4; 3.5 |

Abbreviations: n: number; GC: glucocorticoids; RA: Rheumatoid Arthritis; UA: Undifferentiated arthritis
