# Supplementary material for: A rational use of glucocorticoids in patients with early arthritis has a minimal impact on bone mass
Source: Arthritis Res Ther. 2010 Mar 23;12(2):R50. doi: 10.1186/ar2961 (PMC2888199; doi:10.1186/ar2961)
Supplement: Additional file 3 — Variables associated with glucocorticoid prescription and the cumulative dose of this drug by month of follow-up. Multivariate analysis that provides information about the variables that explain GC prescription and those associated with the cumulative dose of GC. [file ar2961-S3.DOC]

Additional Table 3. Variables associated to glucocorticoid prescription (left columns) and to the cumulative dose of this drug by month of follow-up (right columns).

|  | No GC prescribed | | Cumulative GC dose | |
| --- | --- | --- | --- | --- |
|  | Coef. ± SD | p | Coef. ± SD | p |
| Male  Female | Ref.  1.4 ± 0.7 | 0.041 | Ref  -0.1 ± 0.04 | 0.022 |
| Age (by year) | -0.04 ± 0.02 | 0.032 | 0.01 ± 0.001 | <0.001 |
| Disease duration (by month) | **-** | n.s. | -0.02 ± 0.004 | <0.001 |
| RA  UA | - | n.s. | Ref.  0.12 ± 0.05 | 0.015 |
| DAS 28 v1 | -0.54 ± 0.26 | 0.41 | 0.02 ± 0.01 | 0.083 |
| HAQ v1 | -0.81 ± 0.51 | 0.115 | 0.25 ± 0.03 | <0.001 |
| RF:  - positive  - negative | **-** | n.s. | 0.52 ± 0.03 | <0.001 |
| Anti- CCP:  - positive  - negative | - | n.s. | - | n.s. |
| DMARDs  - no treatment  - monotherapy  - CT | **-** | n.s. | 0.18 ± 0.12  Ref.  0.42 ± 0.04 | 0.116  -  0.051 |

Abbreviations: GC: glucocorticoid; RA: rheumatoid arthritis; UA: undifferentiated arthritis; DAS28: disease activity score in 28 joints; v1: baseline or visit 1; HAQ: health assessment questionnaire; RF: rheumatoid factor; anti-CCP: antibodies directed against cyclic citrullinated peptide; DMARDs: disease-modifying anti-rheumatic drugs; CT: combined therapy.
