# Supplementary material for: A rational use of glucocorticoids in patients with early arthritis has a minimal impact on bone mass
Source: Arthritis Res Ther. 2010 Mar 23;12(2):R50. doi: 10.1186/ar2961 (PMC2888199; doi:10.1186/ar2961)
Supplement: Additional file 4 — Prescription of drugs for osteoporosis and the incidence of fractures during the follow-up. This file provides information about prevalence and variables associated with the prescription of drugs for osteoporosis. In addition, it describes the incidence of clinical fractures in the population described in the article. [file ar2961-S4.DOC]

**Prescription of calcium plus vitamin D and bisphosphonates**

Almost 60% of our patients were prescribed calcium plus vitamin D and 26% of patients also received bisphosphonates at some time during the follow-up period. These drugs were more frequently used in patients with densitometric osteoporosis at baseline, in patients treated with GC and in postmenopausal women (see Additional Table 4 below). We did not find a significant association between bisphosphonate use and the final diagnosis or prior fractures (see Additional Table 4 below).

**Incidence of clinical fractures during follow-up**

During the two-year follow-up there were three new symptomatic fractures. The first was a vertebral fracture in a 68 year-old woman with OP and RA treated with cyclic etidronate. The second case was a 67 year-old man with UA that suffered a peripheral fracture despite displaying a T-score > -1 at all sites and being under prophylactic treatment with calcium plus vitamin D. Finally, a 85 year-old woman with RA and OP who was treated with cyclic etidronate also suffered a peripheral fracture. These three patients had received high cumulative doses of GCs: 88, 189 and 94 mg/month, respectively.

Additional Table 4: Variables associated with the prescription of drugs for osteoporosis in patients with recent onset arthritis

| OP treatment | None (n; %) | Calcium plus vitamin D (n; %) | Bisphosphonates (n; %) | p |
| --- | --- | --- | --- | --- |
| Diagnosis  -RA  - UA | 28; 35.9%  20; 52.6% | 27; 34.6%  11; 28.9% | 23; 29.5%  7; 18.4% | 0.2 |
| Female gender  Male gender | 37; 41.1%  11; 42.3% | 26; 28.8%  12; 46.1% | 27; 30.0%  3; 11.5% | 0.1 |
| Menopause | 14; 27.0% | 12; 23% | 26; 50% | <0.001 |
| BMD at baseline:  - normal BMD  - osteopenia  - osteoporosis | 34; 70.8%  12; 25.0%  2; 4.2% | 23; 60.5%  12; 31.6%  3; 7.9% | 14; 48.3%  12; 41.4%  4; 13.3% | 0.3 |
| Prior fractures | 5; 25.0% | 8; 40% | 7; 35% | 0.2 |
| Use of GC | 17; 25.0% | 27; 39.7% | 24; 35.3% | <0.001 |

Abbreviations: OP: osteoporosis; BMD: bone mineral density; RA: rheumatoid arthritis; UA: undifferentiated arthritis; GC: glucocorticoid.
